# Fibrinogen Alpha Chain Precursor and Apolipoprotein A-I in Urine as Biomarkers for Noninvasive Diagnosis of Calcium Oxalate Nephrolithiasis: A Proteomics Study

**DOI:** 10.1155/2014/415651

**Published:** 2014-07-23

**Authors:** Wei Zhu, Min Liu, Guang-Chun Wang, Bo Peng, Yang Yan, Jian-Ping Che, Qing-Wei Ma, Xu-Dong Yao, Jun-Hua Zheng

**Affiliations:** ^1^Department of Urological Surgery, Shanghai Tenth People's Hospital, Tongji University, Shanghai 200072, China; ^2^Academy for Advanced Interdisciplinary Studies, Peking University, Beijing 100871, China

## Abstract

Calcium oxalate nephrolithiasis is the most common urological disease, but noninvasive and convenient methods of diagnosis are rarely available.* Objective.* The present study aimed to identify potential urine biomarkers for noninvasive diagnosis of CaOx nephrolithiasis.* Methodology.* Urine samples from 72 patients with CaOx nephrolithiasis and 30 healthy controls were collected and proteomics analysis was performed using matrix-assisted laser desorption/ionization-time of flight-mass spectrometer (MALDI-TOF-MS).* Results.* Thirteen proteins/peptides displayed statistically significant differences. The peptides of m/z 1207.23 and 2773.86 were selected by the genetic algorithm (GA) to build a possible diagnostic model. The area under the curve of m/z 1207.23 and 2773.86 was 0.936 and 0.987, respectively. The diagnostic model in distinguishing patients and healthy subjects showed 100% sensitivity and specificity. The peak at m/z 2773.86 was identified as fibrinogen alpha chain (FGA) with the sequence G.EGDFLAEGGGVR.G, and the peak at m/z 2773.86 was identified as apolipoprotein A-I (apoA-I) with the sequence L.PVLESFKVSFLSALEEYTKKLNTQ.* Conclusion.* The study results strongly suggested that urinary FGA and apoA-I are highly sensitive and specific biomarkers for noninvasive diagnosis of CaOx nephrolithiasis.

## 1. Introduction

Nephrolithiasis is a global problem spanning all geographies with an estimated annual incidence of 4% in China and 1-2% in United States of America (USA) [1, 2]. The lifetime risk of nephrolithiasis varies from 1 to 5% in Asia, 10 to 15% in USA, and 5 to 9% in Europe [3]. In addition, recent evidence reveals that renal calculi are becoming more common [4]. According to data from the Urological Diseases in America project, the total annual cost of nephrolithiasis in the USA in the year 2000 was about $5.3 billion. In spite of that, posttreatment recurrence rates are high, and the recurrence rate of nephrolithiasis is expected to exceed 30% within 10 years of an initial stone event [5]. Recent studies showed nephrolithiasis and related obstruction as risk factors to chronic kidney disease (CKD), and CKD was twofold higher among individuals with history of kidney stones [6, 7]. Until now, nephrolithiasis could only be diagnosed by imaging examinations, however, the use of which becomes less when the stone is <5 mm. About 70–80% are calcium-containing stones, which include calcium oxalate (CaOx) and calcium phosphate [8]. CaOx stone may be considered as a complex condition having multiple mechanisms, which have not been clearly understood. Even though a noninvasive diagnostic test for detecting nephrolithiasis has been suggested, there are still few reliable biomarkers that could be used to aid clinicians to diagnose this condition.

In recent years, progress in mass spectrometry and its hyphenation with the separation techniques has made these tools essential in life sciences. MS is a sensitive analytical technique, which is able to quantify analysis and identify unknown molecules. Urine is one of the most important biofluids in clinical proteomics, since it is easily accessible in a large quantity without the use of invasive procedures [9]. In addition, pathophysiologic changes in the urinary tract and kidneys are reflected by changes in the urinary proteome [10]. Coon et al. determined that the human urinary proteome apparently contains over 100,000 different peptides, of which at least 5000 have high frequency [11]. Recent reports have advocated employing the MS approach for determining specific patterns that are indicative of renal, bladder, and prostate cancers [12–14]. Urine is a rich noninvasive source of potential biomarkers of disease that awaits exploration.

The present study aimed to identify potential urine biomarkers for noninvasive diagnosis of CaOx nephrolithiasis by differentiating urinary proteome features between patients with CaOx nephrolithiasis and healthy subjects matrix-assisted laser desorption/ionization-time of flight-mass spectrometer and linear trap quadrupole.

## 2. Materials and Methods

### 2.1. Patients

Patients with nephrolithiasis (*n* = 72; male = 44 and female = 28), who were hospitalized at the Department of Urological Surgery, Shanghai Tenth People's Hospital, between January 2013 and September 2013, were prospectively enrolled in the study. Nephrolithiasis was preoperatively diagnosed in all patients by imaging examination, and they underwent percutaneous nephrolithotomy and retroperitoneal laparoscopic pyelolithotomy and their stone samples were analyzed by Fourier transform infrared spectrometer (Bruker tensor 27, Bruker Germany). Patients with calculi other than CaOx and other diseases such as diabetic nephropathy, CKD, and acute kidney injury were excluded. Thirty healthy medical staff from the institution, who did not have any evidence of nephrolithiasis or other related diseases, were also enrolled as controls. The study protocol was reviewed and approved by the hospital's ethics committee.

### 2.2. Samples Collection

After obtaining informed consent, morning midstream spot urine samples of patients were obtained immediately on the second day after admission and were collected in 50 mL urine cups. Within 4 h, samples were centrifuged at 10000 g for 10 min at room temperature. Aliquots of 800 *μ*L were stored at −80°C until further use. All samples were collected and processed using the same procedure to minimize preanalytical bias.

### 2.3. Proteomics Analysis for the Urine Peptides Differences

MALDI-TOF-MS analysis was performed as recommended in the manufacturer's protocol (Bruker Daltonics, Leipzig, Germany). MALDI-TOF MS samples were processed using a magnetic bead-based weak cation exchanger (WCX, Bruker Daltonics, Germany) according to the manufacturer's protocols. Samples (20 *μ*L each) were diluted with 20 *μ*L binding solution and added to the bead slurry (5 *μ*L) in a 200 *μ*L polypropylene tube, which was mixed thoroughly and incubated in the tube for 10 min. After magnetic bead separation and washings, samples were purified through the following three steps: binding, washing, and elution. The processed samples were analyzed using a linear MALDI-TOF-MS (Ultra-flex; Bruker Daltonics, Leipzig, Germany) equipped with a pulsed ion extraction source. The MALDI-TOF-MS system was controlled by Flex Control software v.2.0 (Bruker Daltonics, Leipzig, Germany). Each spectrum was detected in linear positive mode and was externally calibrated using a mixture of peptide/protein standards between 1000 and 10000 Da.

### 2.4. Identification of Protein Biomarkers by Nanoliquid Chromatography (LC)/Electrospray Ionization- (ESI-) MS/MS

The sequences of differential expression peptides between patients with CaOx nephrolithiasis and controls were identified using a nano-LC/ESI-tandem MS system consisting of an Aquity ultra performance LC system (Waters Corporation, Milford, USA) and LTQ Obitrap XL mass spectrometer (Thermo Scientific, Bremen, Germany) equipped with a nano-ESI source (Michrom Bioresources, Auburn, USA). MS/MS experimental protocol involved the following steps. Peptides were resuspended with 20 *μ*L solvent A (5% acetonitrile, 0.1% formic acid in water). 10 *μ*L peptide solution was loaded onto the Captrap Peptide column (2 mm × 0.5 mm, Michrom Bioresources, Auburn, USA) at a 20 *μ*L/min flow rate of solvent A for 5 min and then was separated on a Magic C18AQ reverse phase column (100 *μ*m id × 15 cm, Michrom Bioresources, Auburn, USA) with a three-step linear gradient: starting from 5% B (90% acetonitrile, 0.1% formic acid in water) to 45% B (in other words, from 95% A to 55% A) in 100 min and increased to 80% B in 3 min and then to 5% B in 2 min. The column was reequilibrated at initial conditions for 15 min. The column flow rate was maintained at 500 nL/min, and column temperature was maintained at 35°C. The electrospray voltage of 1.8 kV against the inlet of MS was used. The MS instrument was operated in a data-dependent model. The range of full scan was 400–10,000 m/z. The eight most intense monoisotope ions were the precursors for collision-induced dissociation. MS/MS spectra were limited to two consecutive scans per precursor ion, followed by 60 s of dynamic exclusion.

The mass spectra were searched using ClinProt Tools software 2.2 (Bruker Daltonik, Leipzig, Germany). Human International Protein Index (IPI) database (IPI human v3.68 fasta with 87061 entries) and National Center for Biotechnology Information (NCBI) protein-protein BLAST database (http://www.ncbi.nlm.nih.gov/BLAST/) were used to search. The searching parameters were set as no enzyme, and oxidation on methionine was set as variable modification. The peptide mass tolerance was 10 ppm, and the fragment ion tolerance was 1.0 Da. Peptides with a confidence level score of >95% were accepted as correct matches.

### 2.5. Diagnostic Model for CaOx Nephrolithiasis

Establishment of model in the training set: the urine peptide profiles of 18 patients with CaOx nephrolithiasis and 6 healthy controls in the training set were analyzed. The reproducibility of mass spectral generation was determined by the mean relative peak intensities. All the spectra obtained from the urine samples in the training set were analyzed using ClinProt Tools to subtract baseline, normalize spectra (using total ion current), and determine peak m/z values and intensities in the mass range of 1000 to 10000 Da. The signal-to-noise ratio should be higher than five. To align the spectra, a mass shift of <0.1% was determined. The peak area was used as quantitative standardization. Comparison of relative peak intensity levels between two groups was also calculated within the software suite.

### 2.6. Statistical Methods and Evaluation of Assay Precision

To evaluate the precision of the assay, within- and between-run variations were determined using multiple analyses of bead fractionation and MS for the two urinary samples. For the within- and between-run variations, three peaks with various intensities were examined. Within-run imprecision was determined by evaluating the coefficient of variation (CV) for each sample using eight assays within a run and between-run imprecision by performing eight assays for 7 d was then determined.

Data obtained by measurements were given as mean ± standard deviation. SPSS (version 17.0, SPSS, Inc, Chicago, IL) was used for the analysis of clinical characteristics of patients with nephrolithiasis and healthy subjects using *χ* ^2^ test or *t*-test. Student's *t*-test was used for analysis of normally distributed continuous data, while Wilcoxon test was used for nonnormally distributed continuous data. Chi-square test was used for categorical data analysis. *P* < 0.05 was considered statistically significant.

## 3. Results

### 3.1. Reproducibility, Precision, and Accuracy

For the precision and accuracy of the proteomic data in the present analyses, the six within-run and six between-run assays reproducibility of the two samples were performed using MALDI-TOF-MS analysis. In each profile, three peaks with different molecular masses were selected to evaluate the precision of the assay. The peak CVs were all <3% in the within-run and <10% in the between-run assays. These values were consistent with the reproducibility data for the protein biology system reported by the manufacturer.

### 3.2. Discovery Screening of Differences between Patients with Nephrolithiasis and Healthy Subjects

**Figure 1:**
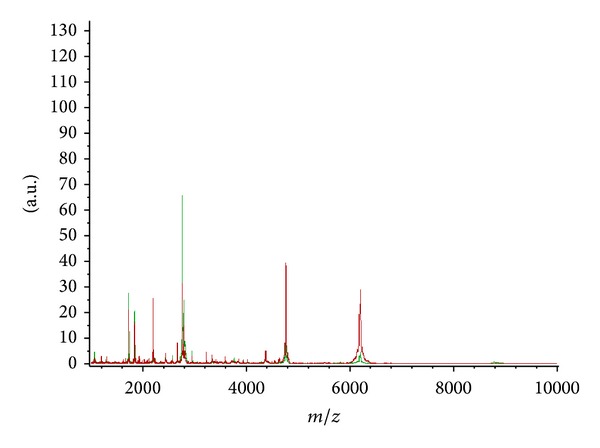
The different mass range of CaOx nephrolithiasis patients (red) and healthy volunteers (green).

**Figure 2:**
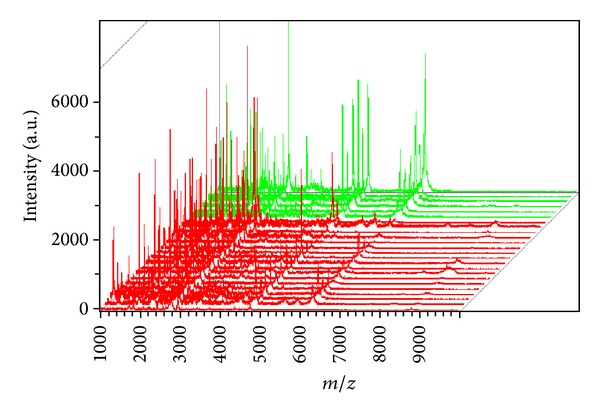
Mass spectrum of the urinary protein profile of the test group obtained by MALDI-TOF-MS (red represents the CaOx and green represents the healthy).

To screen urine peptides of interest to diagnose CaOx nephrolithiasis, urinary samples of training set from 54 patients with CaOx nephrolithiasis and 24 healthy controls were analyzed by MALDI-TOF-MS with WCX-MB (Table 1). Samples were randomly distributed during processing and analysis. A total of 56 distinct m/z values were resolved in the 1000 to 10000 Da range (Figure 1). Differences in peak positions and intensities were observed, and two-tailed *t*-test was used to obtain a *P* value for each peak and rank the peaks with *P* value (Figure 2). The result of *t*-test showed that 13 proteins/peptides (including 8 downregulated and 5 upregulated proteins/peptides) displayed statistically significant differences (*P* < 0.05) between patients with CaOx nephrolithiasis and healthy subjects (Table 2).

### 3.3. Selection of Urinary Biomarker and Assessment of Diagnostic Efficacy

A class prediction model set up by genetic algorithm (GA) in ClinProTools was utilized to generate diagnostic models to discriminate patients with CaOx nephrolithiasis from the healthy controls in training set. Among the differentially expressed proteins/peptides, m/z 1207.23 and m/z 2773.86 were selected by the GA to build a possible diagnostic model. The peptide of m/z 1207.03 was observed to be significantly downregulated in patients with nephrolithiasis (*P* = 0.00856). On the contrary, the peptide of m/z 2773.86 was observed to be remarkably upregulated in the patients (*P* = 0.00867).

**Figure 3:**
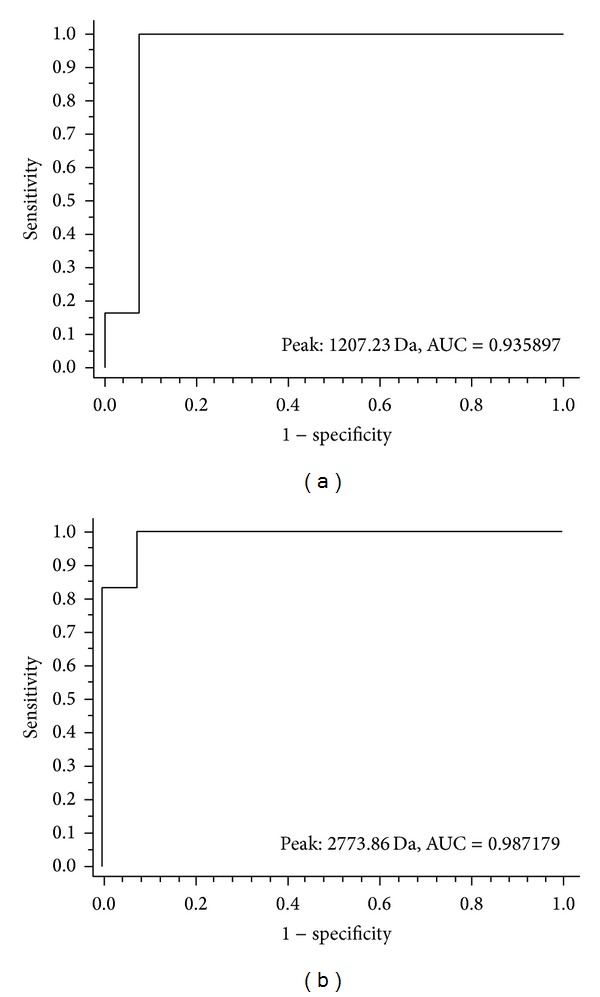
Receiver operating characteristic (ROC) curve of the two peptides (m/z 1207.23 and 2773.86) selected for the diagnostic model of CaOx. (a) and (b) represent the ROC of peptides at m/z 1207.23 and 2773.86 (AUC, areas under the ROC curve).

The accuracy of the established GA classification model in the testing was verified, which included 18 patients with CaOx nephrolithiasis and six healthy volunteers. The diagnostic capability of each peak was determined by the receiver operator characteristic (ROC) curve. When the peptide of m/z 1207.03 was used to distinguish patients from the healthy subjects, the area under the curve (AUC) of m/z 1207.03 was 0.936 (a sensitivity of 94.4% and specificity of 83.3%, 95% CI: 0.909 to 0.962), and the AUC of m/z 2773.86 was 0.987 (a sensitivity of 94.4% and specificity of 100%, 95% CI: 0.925 to 0.996) (Figure 3, Tables 3 and 4). The GA model established with the two peaks defining correct which classified the nephrolithiasis samples as positive and healthy samples as negative provided both 100% sensitivity and specificity in the testing.

### 3.4. Identification of Markers

**Figure 4:**
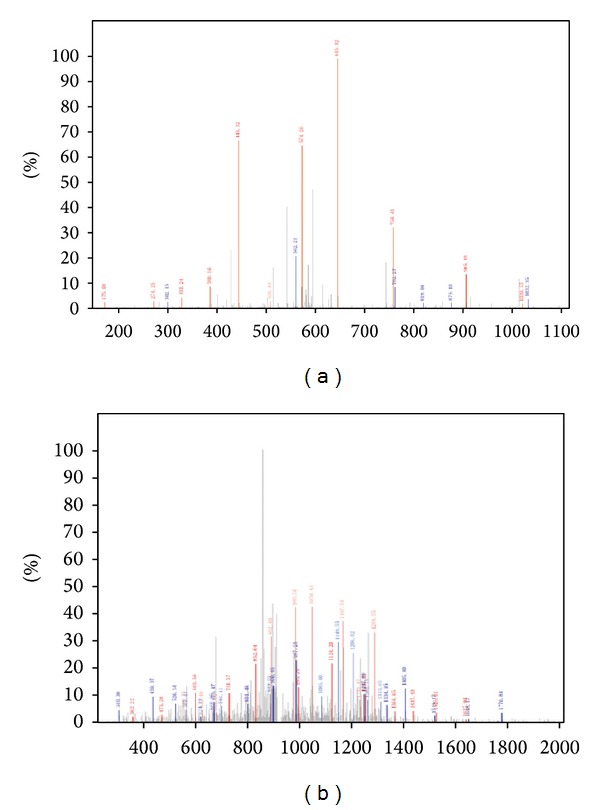
MS/MS identification of urine peptides fibrinogen alpha chain (a) and apolipoprotein A-I (b).

The potential protein peptides at m/z 1207.03 and m/z 2773.86 could distinguish patients with CaOx nephrolithiasis from the healthy subjects. With LTQ-Orbitrap-MS detection, 6 peptides (including m/z 1207.03 and m/z 2773.86 Da) among the total 13 peptides of differential expression were identified successfully, and others were uncharacterized (Table 5). The MS data was subjected to IPI human v3.68 fasta with 87061 entries for peptide sequencing and NCBI database for protein identification. The peptide of m/z 1207.03 was identified as fibrinogen alpha chain precursor with the sequence G.EGDFLAEGGGVR.G (IPI00021885, gene symbol = FGA) (Figure 4(a)). The peak at m/z 2773.86 was identified as apolipoprotein A-I precursor with the sequence L.PVLESFKVSFLSALEEYTKKLNTQ.-(IPI00021841, gene symbol = apoA-I) (Figure 4(b)).

## 4. Discussion

Urolithiasis is a relevant clinical problem with a subsequent burden for one's health. With its complex etiology and high rate of recurrences, urinary tract stone disease possesses a medical challenge. Calcium containing stones are the most common, constituting about 75% of all urinary calculi. Under normal examinations such as blood and urinary tests, urologists could not make precise diagnosis until the identification of renal function injury and inflammation. On the contrary, the imaging tests such as X-ray and computed tomography scan are widely used to diagnose calculi of even a small size. However, the radiographical examinations are not suitable for children, teenagers, and pregnant women due to chances for the development of radiation-induced injury. Application of noninvasive and convenience methods to diagnose and assess renal stone disease is still a challenge to clinicians.

Many proteins present in stone and some proteins normally present in urine, but their role in urolithiasis remains unknown. Developments in proteomics have successfully been applied to meet the demands. Proteomic analyses based on MS technique provided us the innovative ways to identify the components of urinary protein complexes. Proteomic profiling is based on the fact that proteins represent the dynamic state of the cells, reflecting earlier pathological and physiological changes in the disease more accurately than genomic sequencing [15]. Numerous studies have widely recognized the value of proteomics as a diagnostic and predicting tool in various diseases [16, 17]. Urine is both easily available in large quantities without invasive procedures and also stable enough for proteome analysis [18]. Urinary proteomic analysis has shown that several renal diseases can be detected by the presence of specific polypeptides in the urine [19, 20]. The recent proteomic studies of nephrolithiasis have revealed the components of stone. Aggarwal et al. [21] identified three antilithiatic cationic proteins such as histone-lysine N-methyltransferase, inward rectifier K channel, and protein Wnt-2 by MALDI-TOF-MS in CaOx stones. However, only few studies have focused on the difference in urinary proteome between the patients with CaOx nephrolithiasis and healthy subjects.

In this study, a case-control comparative analysis was designed between patients with CaOx nephrolithiasis and healthy subjects, and the difference in the urinary proteome was analyzed. The protein profiling was performed by MALDI-TOF-MS after proteome fractionation with magnetic beads, and it acts as the precise and rapid technique for investigation of complex urine samples. The urine samples of patients with CaOx nephrolithiasis showed 13 significantly differentiated proteins/peptides, including 8 downregulated and 5 upregulated proteins/peptides. The most two significantly differentiated peptides, which are obtained at m/z 1207.03 and m/z 2773.86, were developed as diagnosis model by the GA analysis. The diagnosis model achieved recognition capacity and cross validation close to 100% to discriminate patients with CaOx nephrolithiasis from the healthy subjects. The AUC of peak at m/z 1207.03 and m/z 2773.86 Da determined by the ROC curve were 0.936 and 0.986. Further, m/z 1207.03 and m/z 2773.86 Da were identified as fibrinogen alpha chain precursor (FGA) and apolipoprotein A-I precursor (apoA-I) by LTQ Obitrap XL.

FGA fragments were observed at m/z 1207.23 and m/z 2847.86, respectively, and the m/z 1207.23 suggested high sensitivity and specificity in differentiating the patients with CaOx nephrolithiasis and healthy subjects. FGA has been identified in urine to assist diagnosis and evaluate prognosis in many diseases such as urinary tract infection [22], bladder cancer [23], and kidney ischemia/reperfusion injury [24]. FGA is a protein, which is encoded in human by the FGA gene, and it is the component of fibrinogen (FG), a major blood protein that consists of pairs of three different polypeptide chains such as *α*, *β*, and *γ*, joined by disulfide bonds to form a symmetric dimeric structure. Fibrinogen can be digested either by plasmin or by thrombin, and the activities of plasmin and thrombin are regulated or progressively activated by calcium ions; therefore, the changes in the calcium ion channels in the CaOx calculi may affect the fibrinolytic system. In addition, Drew et al. [25] found that fibrinogen plays an important role in the progress of tissue repair. Fg-deficient (Fg^−/−^) mice were studied in a cutaneous wound healing model, and the results revealed an abnormal pattern of tissue repair including misguided epithelium, delayed wound closure, and reduced tensile strength. In the present study, FGA was downregulated in patients with CaOx nephrolithiasis, which suggested that the low level of FGA might lead to abnormal nephron repair and stone formation.

The results of the present study have suggested that a high level of urine apoA-I may be associated with CaOx nephrolithiasis. ApoA-I constitutes approximately 70% of the apolipoprotein content of high-density lipoprotein particles, and several studies have reported the apoA-I changes in kidney diseases. Sethi et al. [26] analyzed the renal biopsy and nephrectomy specimens of renal amyloidosis and identified the expression of apoA-I in the amyloid deposits. Lopez-Hellin et al. [27] reported that apoA-I was remarkably increased in the urine of patients with focal segmental glomerulosclerosis. The kidney cortex appears to be an important site of apoA-I catabolism. This uptake has been thought to be the result of glomerular filtration, tubular reabsorption, and intracellular degradation of lipid-poor apoA-I [28]; and the progress is caused by the binding of apoA-I to cubilin, functioning in line with megalin. Cubilin has been shown to form a functional receptor complex with the protein [29]. Kozyraki et al. found apoA-I loss in the urine of humans with defective cubilin and cubilin-amnionless function [30]. Hence, it could be speculated that the aopA-I upregulation in urine of patients with CaOx may be because of the reason that calculi affect the normal function of glomerular and break the balance of cubilin-amnionless. However, the association of changes in aopA-I levels and differences in the lipoprotein-related proteome between the urine of patients with CaOx nephrolithiasis and healthy controls remains unclear and warrants further in-depth investigation.

The FGA and apoA-I proteins/peptides could serve as diagnosis-associated biomarkers for patients with CaOx nephrolithiasis. Moreover, they may contribute to the establishment of a novel noninvasive diagnostic method and may facilitate personalized medical therapy for patients with CaOx nephrolithiasis. Further proteomics researches are recommended to identify the diagnostic difference of peptide fragments used in detecting different sorts of calculi.

## Figures and Tables

**Table 1:** General information of CaOx nephrolithiasis patients and healthy volunteers.

Characteristics	Training set	Test set	Total
Nephrolithiasis			
Number of cases (*n*)	54	18	72
Gender (M/F)	32/22	12/6	44/28
Mean age (years)	33.5 ± 4.9	30.4 ± 5.3	32.9 ± 5.0
Stone size (length × width × height/mm)	18 ± 6 × 10 ± 1.5 × 14 ± 6	11 ± 3 × 7 ± 1 × 10 ± 2^a^	15 ± 4 × 9 ± 1 × 12 ± 4
Grade of hydronephrosis			
I	19	4	23
II	30	6	36
III	10	3	13
Blood urea nitrogen (mmol/L)	5.5 ± 1.2	4.9 ± 0.9^a^	5.2 ± 1.4
Serum creatinine (*μ*mol/L)	87.19 ± 7.98	79.41 ± 9.16^a^	84.74 ± 6.98
Volunteers			
Number of cases (*n*)	24	6	30
Gender (M/F)	17/7	4/2	21/9
Mean age (years)	25.6 ± 3.7	27.2 ± 1.8	26.1 ± 2.5
Blood urea nitrogen (mmol/L)	4.8 ± 0.4	4.2 ± 1.5^b^	4.5 ± 0.9
Serum creatinine (*μ*mol/L)	66.91 ± 5.49	62.43 ± 4.17^b^	64.89 ± 4.88

^a^ *P* > 0.05, test set compared with training set in CaOx nephrolithiasis

^b^ *P* > 0.05, test set compared with training set in healthy volunteers.

Standard BUN 1.8–7.1 mmol/L, standard SCr 59–104 mmol/L.

**Table 2:** Distribution of *P* value-specific markers between CaOx nephrolithiasisand healthy control.

m/z^a^	MRI (SD)^b^ in nephrolithiasis	MRI (SD)^b^ in healthy control	Regulation in nephrolithiasis	*P* value^c^
1207.23	3.64 (1.82)	7.85 (1.12)	↓	0.00856
2773.86	83.95 (28.39)	27.53 (15.98)	↑	0.00867
6182.87	144.19 (66.93)	38.66 (17.53)	↑	0.00878
2847.86	93.37 (67.89)	517.79 (105.24)	↓	0.0012
6295.88	112.66 (79.63)	647 (152.98)	↓	0.00168
4754.38	202.07 (169.92)	603.03 (148.77)	↓	0.00216
6131.83	49.73 (22.97)	202.03 (46.99)	↓	0.00216
6083.70	15.22 (5.15)	53.19 (12.59)	↓	0.00302
6069.56	15.7 (5.36)	48.95 (11.46)	↓	0.00302
2830.18	54.1 (23.42)	22.1 (9.66)	↑	0.00325
6327.86	6.39 (2.26)	20.96 (5.87)	↓	0.00642
2757.24	389.82 (183.01)	172.63 (111.86)	↑	0.0279
2726.01	34.08 (12.71)	19.42 (7.81)	↑	0.0314

^a^m/z, average mass.

^b^MRI, mean relative intensity.

^c^ *P* value calculated with *t*-test. Statistical significance was considered at *P* < 0.05.

**Table 3:** Predictive values based on urine FGA protein.

Group	Test	Sensitivity (94.4%)	Specificity (83.3%)
Nephrolithiasis	18	17/18	
Healthy control	6		5/6

**Table 4:** Predictive values based on urine apoA-I.

Group	Test	Sensitivity (94.4%)	Specificity (100%)
Nephrolithiasis	18	17/18	
Healthy control	6		6/6

**Table 5:** Identification of sequences of peptide differentially expressed between CaOx nephrolithiasis and healthy control.

m/z^a^	Peptide name	Peptide sequences
1207.23	FGA isoform 1 of fibrinogen alpha chain precursor	G.EGDFLAEGGGVR.G
2773.86	apoA-1 apolipoprotein A-I precursor	L.PVLESFKVSFLSALEEYTKKLNTQ.-
6182.87	Uncharacterized peptide	
2847.86	FGA isoform 1 of fibrinogen alpha chain precursor	S.SSYSKQFTSSTSYNRGDSTFESKSY.K
6295.88	Uncharacterized peptide	
4754.38	Uncharacterized peptide	
6131.83	Uncharacterized peptide	
6083.70	Uncharacterized peptide	
6069.56	Uncharacterized peptide	
2830.18	TMSL3 thymosin beta-4-like protein 3	K.KTETQEKNPLPSKETIEQEKQAGES.-
6327.86	Uncharacterized peptide	
2757.24	F13A1 coagulation factor XIII A chain precursor	R.RAVPPNNSNAAEDDLPTVELQGVVPR.G
2726.01	COPA coatomer subunit alpha	R.FWVLAAHPNLNLFAAGHDGGMIVFK.L

^a^m/z, average mass.
